# An overview of systematic reviews on upper extremity outcome measures after stroke

**DOI:** 10.1186/s12883-015-0292-6

**Published:** 2015-03-11

**Authors:** Margit Alt Murphy, Carol Resteghini, Peter Feys, Ilse Lamers

**Affiliations:** Institute of Neuroscience and Physiology, Rehabilitation Medicine, Sahlgrenska Academy, University of Gothenburg, Per Dubbsgatan 14, 3tr, S- 413 45 Göteborg, Sweden; School of Health, Sport and Bioscience, University of East London, London, UK; REVAL—Rehabilitation Research Centre, BIOMED—Biomedical Research Institute, Faculty of Medicine and Life Sciences, Hasselt University, Hasselt, Belgium

**Keywords:** Upper limb, Psychometrics, Rehabilitation, Outcome assessment (Health Care) outcome measures, Review

## Abstract

**Background:**

Although use of standardized and scientifically sound outcome measures is highly encouraged in clinical practice and research, there is still no clear recommendation on which tools should be preferred for upper extremity assessment after stroke. As the aims, objectives and methodology of the existing reviews of the upper extremity outcome measures can vary, there is a need to bring together the evidence from existing multiple reviews. The purpose of this review was to provide an overview of evidence of the psychometric properties and clinical utility of upper extremity outcome measures for use in stroke, by systematically evaluating and summarizing findings from systematic reviews.

**Methods:**

A comprehensive systematic search was performed including systematic reviews from 2004 to February 2014. A methodological quality appraisal of the reviews was performed using the AMSTAR-tool.

**Results:**

From 13 included systematic reviews, 53 measures were identified of which 13 met the standardized criteria set for the psychometric properties. The strongest level of measurement quality and clinical utility was demonstrated for Fugl-Meyer Assessment, Action Research Arm Test, Box and Block Test, Chedoke Arm and Hand Activity Inventory, Wolf Motor Function Test and ABILHAND.

**Conclusions:**

This overview of systematic reviews provides a comprehensive systematic synthesis of evidence on which outcome measures demonstrate a high level of measurement quality and clinical utility and which can be considered as most suitable for upper extremity assessment after stroke. This overview can provide a valuable resource to assist clinicians, researchers and policy makers in selection of appropriate outcome measures.

**Electronic supplementary material:**

The online version of this article (doi:10.1186/s12883-015-0292-6) contains supplementary material, which is available to authorized users.

## Background

Stroke is a major cause of long-term disability worldwide [1]. Motor impairments of the upper extremity are common and affect approximately 50-70% of patients in the acute [2-4] and 40% in the chronic phase [5,6]. A person’s ability to perform everyday tasks, to participate in the society and the quality of life can be significantly compromised after stroke [7].

Evaluation of the effectiveness of rehabilitation interventions after stroke is highly prioritized and encouraged in stroke guidelines and policies. Despite consensus among nationally published guidelines recommending the use of valid and reliable assessment tools, further direction does not extend to which outcome measures (OM) should be selected for particular evaluative needs [8-11]. However more recently, two published physiotherapy guidelines, the KNGF Clinical Practice Guideline for physical therapy in patients with stroke [12] and the Neurology section of the American Physical Therapy Association (StrokeEdge task force) [13] have provided more specific recommendations. This suggests the availability of appropriate evidence for extended evaluation and synthesis. Indeed, during the last decade, numerous studies focusing on upper extremity OM have been published, many highlighting the need of standardized definitions and higher consensus and guidance in OM selection [14-16]. A more uniform reporting of OM in stroke studies would allow comparison across studies and enable pooling of data from different studies for evidence synthesis. One example on improving standardization of outcomes across several research areas is the COMET (Core Outcome Measures in Effectiveness Trials) initiative, which aims to improve development and application of agreed standardized sets of outcomes, the “core outcome sets” [17]. This initiative has recently launched a database currently containing more than 500 references, but only a few of these target the stroke population and upper extremity function is not yet covered [18,19]. The OM assessing the arm and hand function are, however, often included in studies and have shown to be the second largest category of OM used in randomized clinical trials after activities of daily living measures [20].

The aims and objectives for systematic reviews of upper extremity OM can vary. For example, a review can only include OM to evaluate a specific type of intervention (e.g. robot-assisted trials) [14,21] or only identify OM reflecting real life function [22]. The majority of the reviews evaluate the psychometric properties and clinical utility of OM. Others focus on the process of OM selection involving participation by clinicians, management and policy makers, and researchers [21,23]. In addition, differences can exist between reviews regarding study inclusion, appraisal process and methodology. These variables make it more difficult for clinicians, researchers and decision makers to determine which measures should be selected to evaluate outcome, to facilitate clinical decision making or to make a valid long-term prognosis.

As more systematic reviews are published, the potential to systematically compare integrate and synthesize the findings increases. Recently, this kind of evidence synthesis on reviews, known as overviews of systematic reviews, has become more common [24-27]. An overview on reviews requires similar search strategy and quality assessment as systematic reviews of primary literature, but relies on the findings reported by the reviewers rather than appraising the primary sources. This approach allows synthesis of the evidence, where comparison and contrasting of the findings from single reviews becomes possible and the identification of existing gaps or trends in the literature more visible. Overviews of systematic reviews can serve as an important source of information for focused communication in the identification of OM that could be included into the clinical practice stroke guidelines.

Currently, the European Network on Robotics for Neurorehabilitation, funded by the European Co-operation in science and technology (COST) action is developing guidelines and evidence-based recommendations for upper extremity assessment in neurological conditions. This overview will be part of these guidelines focusing on available evidence for upper extremity outcome measures that are recommended for use in clinical practice and research. As part of the COST Action, we sought to establish the general state of knowledge in the area through a structured overview of systematic reviews. The aim of this overview is to identify all relevant systematic reviews evaluating upper extremity outcome measures in people with stroke and provide a synthesis of evidence regarding the psychometric properties and clinical utility of the recommended outcome measures.

## Method

### Search strategy

A systematic search of the literature was performed independently by two investigators using electronic databases of PubMed, CINHAL, Cochrane Library, Pedro, NICE (National Institute for Clinical Effectiveness, includes MEDLINE, EMBASE and CINHAL). The initial search strategy was constructed for PubMed and adapted to other databases. A combination of MeSH terms and key words entered at three levels was used: (stroke OR hemiparesis OR hemiplegia*) AND (“upper extremity” OR “upper limb” OR arm) AND ("outcome assessment" OR “outcome measure” OR outcome* OR measure* OR instrument* OR scale* OR test* OR questionnaire*). To narrow the search following search limitations were used: systematic review, review, abstract available, publication date from 2004/01/01 to 2014/02/20, humans, English language, adult: 19+ years, field: title and abstract. In addition, the lists of related articles of the included records from the Pubmed search were screened.

**Figure 1:**
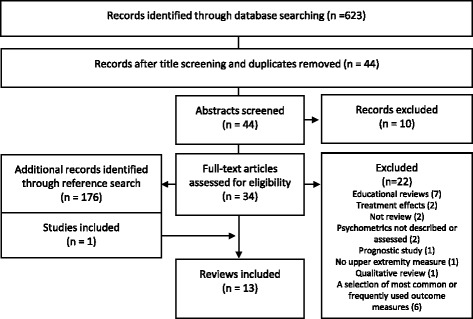
**Flowchart over the search strategy and article selection process (according to the PRISMA guidelines).**

Identification of relevant articles including initial screening of titles and abstracts, selection of relevant articles for the full-text screening and final inclusion were all performed independently by two authors. Inclusion of articles was based on the agreement between the two independent reviewers. When the decision on inclusion was not clear on the basis of the title or abstract, studies were selected for further full text screening. The references of the articles included for the full text screening were also hand searched for additional identification of relevant records, by one investigator and this list was checked by the second investigator. A flow chart of the inclusion process is displayed in Figure 1.

Inclusion criteria for reviews:

- systematic reviews published in peer-reviewed journals,
- involving participants with stroke and/or hemiparesis due to stroke,
- reporting a clear objective to identify outcome measures specific for the upper extremity and/or include measures with a specific separate subsection for the upper extremity,
- report on and/or evaluate the psychometric properties of the outcome measures,
- participants older than 18 years,
- publication year 2004 or later (up to February 2014)

Exclusion criteria for reviews: reviews investigating effectiveness of interventions or treatments, monitoring recovery, focusing on diagnostic screening or prognosis, educational or state-of-the-art reviews, clinical commentaries, case reports, non-structured reviews, descriptive reviews, qualitative reviews, non-human studies. The grey literature (thesis, reports, conference proceedings, government documents, policy documents etc.) was not included. This overview aimed to identify reviews that intended to include all available instruments for measuring a particular construct (upper extremity functioning) and therefore, reviews including a single specific instrument or a selection of most common or frequently used instruments were excluded. The PRISMA guidelines have been followed when applicable (www.prisma-statement.org). No prior protocol has been published for this overview and no financial support was sought or received for the data analysis or drafting of this manuscript. Ethical approval was not applicable.

### Quality assessment and data extraction

The critical appraisal and data extraction of the included systematic reviews comprised: (1) methodological quality assessment of the review process, (2) extraction of descriptive information, (3) extraction of upper extremity outcome measures included in the reviews, (4) identification of OM meeting the standards and criteria for psychometric properties as described by the authors of the reviews, (5) extraction and integration of information on psychometric properties and clinical utility for the final set of outcome measures.

There is no specific assessment tool or checklist available for appraisal of methodological quality of systematic reviews examining clinical outcome measures and their measurement properties. In this overview the AMSTAR (Assessment of Multiple Systematic Reviews) quality assessment tool including 11 items was used as primary guideline for this process [26,28,29]. AMSTAR contains items on the quality of search strategy, article selection, data extraction, appraisal of the scientific quality of the included studies and how the findings and conclusions are reported. All included reviews were screened by two investigators independently using the AMSTAR tool. Disagreements related to the quality assessment were resolved through discussion and, if required, a third reviewer was consulted.

Two investigators independently extracted descriptive data from the included reviews including the publication years, primary objective, research questions, target population, outcome measures (OM), standards and/or criteria used for evaluation of measurement properties of outcome measures. Firstly, all OM included in the reviews targeting upper extremity function after stroke were extracted. Secondly, the OM meeting the criteria or standards for the psychometrics were identified (provided by the authors of the reviews). Standards refer to a defined guideline or clear definition on study design and methods used for evaluation of the measurement properties in the primary studies. Criteria indicate a pre-set cut-off or level that is considered to indicate adequacy for a good measurement property. If no clear standard was reported in the review, the minimum criteria for psychometrics were set to: at least adequate reliability and validity as well as reported evaluation of responsiveness or amount of change as defined by the Sivan et al. [14]. The OM that met these criteria composed the final set of measures from which information on psychometric properties and clinical utility, as reported in the reviews, was summarized. The clinical utility included two aspects: time to administer and administration burden (administration, scoring, interpretation and cost). The data extraction was performed independently by two investigators and the information was compared. Discrepancies were resolved through discussion and a third person consulted if required.

## Results

### Description of included reviews

The literature search process yielded 623 records of which 13 met the inclusion criteria of this overview on systematic reviews (Table 1). The search process and reasons of exclusion of the full text articles are provided in the Figure 1 (for reasons of exclusion for each full text article see Additional file 1). Nine of the included reviews targeted individuals with stroke, and four incorporated stroke condition as part of a wider population search (neurologic conditions, adults in community rehabilitation, individuals with spasticity or upper extremity impairments stroke). All reviews used the International Classification of Functioning, Disability and Health (ICF) as a framework for classifying the outcome measures under different domains. Four reviews incorporate measures at all levels of ICF, one review included measures at impairment level, four at the activity level and 3 on both; and one review searched for measures at the participation level of the ICF alone. Some reviews had a distinct search area including only measures, for example, reflecting the “real-life” function, or used in studies evaluating training with robotic devices [14,21] or accelerometry [30]. One review incorporated the development of clinical practice guidelines for physiotherapy and one review had the clinical utility as important criteria. More detailed information on description of the included reviews is displayed in Tables 1 and 2.

**Table 1:** **Methodological quality assessment of the included systematic reviews**

**Author year**	**Primary objective or research question**	**Literature search and data extraction process**				**Requirements of the measurement properties for the outcome measures in primary studies**		
		**Comprehensive search (>2 databases, strategy)/number of papers included**	**Clear inclusion/exclusion criteria**	**Duplicate search**	**Duplicate data extraction**	**Reference provided**	**Values reported**	**Standard and/or criteria**
Ashford 2008 [22]	Identify valid and reliable OM (real-life function)	Yes/84	Yes	Yes	Yes	Yes	Level	Standard & criteria
Baker 2011 [21]	Selection strategy and identification of scientifically sound UE OM suitable for robot trials	Yes/230	Yes	-	-	Yes	Partly	Standard
Connell 2012 [31]	Review psychometrics and clinical utility of UE OM	Yes/NR	Yes	Yes	Yes	Yes	Yes	Standard & criteria
Croarkin 2004 [32]	Review and evaluate psychometrics of UE motor function tests	Yes/170	Yes	Yes	-	Yes	Yes	Standard & criteria
Gebruers 2010 [30]	Assess psychometrics and clinical applicability of accelerometry measures	Yes/25	Yes	Yes	-	Yes	yes	No
Hillier 2010 [23]	Develop and evaluate a process of OM selection for community settings	Yes/300	Yes	-	20%	No	No	Standard
Lemmens 2012 [33]	Identify, evaluate, categorize valid and reliable activity level UE OM	Yes/747	Yes	Yes	-	Yes	No	No
Platz 2005 [34]	Review evidence of psychometric properties of OM for spasticity	Yes/110	Yes	Yes	Yes	Yes	Partly	No
Simpson 2013 [35]	Review the responsiveness of OM for UE recovery	Yes/68	Yes	-	-	Yes	Yes	No
Sivan 2011 [14]	Classify, evaluate UE OM used in robot-assisted trials	Yes/28	Yes	Yes	Yes	Yes	Level	Standard & criteria
Tse 2013 [36]	Identify, evaluate the psychometrics of participation OM	Yes/119	Yes	-	-	Yes	Yes	Standard & criteria
Van Peppen 2007 [37]	Develop clinical practice guideline for physiotherapy (OM, intervention, prognosis)	Yes/32	Yes	Yes	Yes	Yes	Yes	Standard & criteria
Velstra 2011 [38]	Review reliability, responsiveness and content validity of UE OM	Yes/44	Yes	Yes	-	Yes	No	Not reported

*Abbreviations*: *OM* outcome measures, *UE* upper extremity.

**Table 2:** **Overview of the measurement level, target population, upper extremity outcome measures included in the reviews and recommended or meeting the criteria of psychometrics as reported in the primary reviews**

**Author year**	**ICF level (special interest)**	**Target population**	**OM for UE and stroke (number)**	**List of included OM for UE**	**Recommended or met the criteria of psychometrics**		
					**Number**	**OM**	**Standard or criteria used**
Ashford 2008 [22]	Activity (real-life functioning)	stroke, brain injury	6	MAL (12,14, 26, 28 items), ABILHAND, Leeds Adult Spasticity Impact Scale	1	*ABILHAND*	Met 9 of 11 criteria
Baker 2011 [21]	Body function and activity (robot-assisted trials)	stroke	25	ARAT, CAHAI, 10s test, AMAT, WMFT, FMA, MSS, MAS, DeSouza, RMA, STREAM, MESUPE, MI, NHPT, FAT, Sodring Motor Evaluation Test, Sollerman, MCA, MMAC, BBT, Functional Test; Patient-reported: DHI, MAL, ABILHAND, UMAQS	3 Additional scales (2)	*CAHAI, STREAM, ABILHAND* Additional (FMA, ARAT)	MOT and FDA standards; psychometrics provided for CAHAI, STREAM, ABILHAND
Connell 2012 [31]	Body function and activity (clinical utility)	neurologic conditions	11	BBT, NHPT, ARAT, ABILHAND, MAL (14,26), RMA, MSS, Sollerman, Simplified STREAM, Fitts Reaching test	2	*BBT, ARAT*	Clinical utility criteria of ≥8; criteria of validity, intra/inter-rater reliability, ability to detect change
Croarkin 2004 [32]	Body function and functional limitation (no disability scales)	stroke	9	ARAT, CMSA, FMA, MMAC, MAS, MCA, MI, NHPT, RMA	6	*NHPT, FMA, MI, CMSA, ARAT, MAS*	Met 2 of 3 criteria: validity, inter-rater, test-retest reliability; psychometrics provided
Gebruers 2010 [30]*	Activity (accelerometry)	stroke	NA	Accelerometry	NA	NA	No specific criteria; psychometrics provided
Hillier 2010 [23]	ICF (clinical use)	stroke	7	Manual muscle testing, Tardieu Scale, WMFT, Grip strength, CAHAI, Hand Active Sensation Test, NHPT	2	*CAHAI*	Standards described for reliability, validity, responsiveness, utility; psychometrics not provided
Lemmens 2012 [33]*	Activity	stroke, CP	17	AMAT, CAHAI, FAT, TEMPA, ARAT, JHFT, MESUPE, WMFT, ABILHAND, MAL, DHI, UBDS, Actual amount of use, Functional test, MFT, Hand Function Survey, Accelerometry	9	AMAT, CAHAI, FAT, UBDS, ARAT, JHFT, WMFT, DHI, MAL-26	No specific criteria; reference provided for validity, reliability, responsiveness; psychometrics not provided
Platz 2005 [34]*	Body function (spasticity)	stroke, MS, SCI and CP with spasticity	11	Ashworth Scale (original, modified, velocity corrected), Muscle Tone Scale, Modified Tardieu Scale, VAS for tone, Tone assessment Scale, ROM (goniometer, estimation), Finger curl test, Tendon reflex scale	0	-	No specific criteria; psychometrics not provided
Simpson 2013 [35]*	Activity (responsiveness)	stroke	14	ABILHAND, AMAT, ARAT, Accelerometry, CAHAI, DHI, FAT, Functional Test, Hand Function Survey, JHFT, MAL, SIS, TEMPA, WMFT	5	ABILHAND, ARAT, MAL, SIS, WMFT	No specific criteria; MCID values provided
Sivan 2011 [14]	ICF (robot-assisted trials)	Stroke	17	FMA, MSS, CMSA, Ashworth Scale, MRC, Kinematics, Grip strength, NHPT, BBT, ARAT, WMFT, CAHAI, AMAT, RMA (arm), FAT, MAS, ABILHAND	5	*FMA, Kinematics, ARAT, WMFT, ABILHAND*	Criteria high/excellent/moderate for validity, reliability, responsiveness provided
Tse 2013 [36]	Participation	stroke	1	SIS	0	**-**	Criteria for reliability, internal consistency, validity
van Peppen 2007 [37]	ICF (clinical utility for physiotherapy practice)	stroke	10	Core set: MI, FAT; Optional: ROM, Numeric Pain Rating Scale, Nottingham Sensory Assessment, Modified Ashworth Scale, FMA, Hand volumeter, ARAT, NHPT	2	*MI, FAT (core set)*	Level of evidence at least 2 (psychometric properties, clinical utility, ICF)
Velstra 2011 [38]*	ICF (reliability, responsiveness)	stroke, tetraplegia, peripheral or reumathology conditions	8	Ashworth Scale, ARAT, MAL, WMFT, JHFT, FMA, Muscle strength, ROM	2	ARAT, MAL	No specific criteria; grading very good/good for reliability, internal consistency and responsiveness provided; psychometrics not provided

*Reviews did not provide standards, criteria or sufficient information on psychometrics needed for qualification; OM printed in italic were included into the final set (n = 13); *Abbreviations*: *ICF* International Classification of Functioning, Disability and Health, *OM* outcome measures, *UE* upper extremity, *MS* Multiple sclerosis, *SCI* spinal cord injury, *CP* cerebral palsy, *CAHAI* Chedoke Arm Hand Activity Inventory, *STREAM* Stroke Rehabilitation, Assessment Movement, *BBT* Box and Block Test, *ARAT* Action Research Arm Test, *NHPT* Nine Hole Peg Test, *FMA* Fugl-Meyer Assessment (motor), *MI* Motoricity Index, *CMSA* Chedoke-McMaster Stroke Assessment, *MAS* Motor Assessment Scale, *MAL* Motor Activity Log, *SIS* Stroke Impact Scale, *WMFT* Wolf Motor Function Test, *FAT* Frenchay Arm Test (abbreviations for all OM are provided in the end section of the paper).

### Assessment of the methodological quality

Table 1 summarizes the methodological quality of the reviews and requirements of the psychometric properties for the OM as reported by the authors of the reviews. All reviews presented a clear aim and stated specific inclusion and exclusion criteria for identifying the articles and outcome measures for inclusion and data extraction, but no review referred to a published protocol regarding the “a priori” design. The majority of the reviews reported duplicate study selection and half of them also employed duplicate data extraction process. Four reviews demonstrated a comprehensive and systematic literature search and data extraction along with clearly defined standards and criteria requirements on measurement properties of the outcome measures [14,22,31,37]. Two reviews defined a limitation of sample size (>10) in inclusion criteria [35,38]. Eleven reviews discussed possible limitations concerning the publication bias, but only six reviews provided a clear conflict of interest statement. All reviews reported the number of included articles, but only two provided a full list of included studies [14,30]. All reviews listed and reported the number of included outcome measures, and six provided references or a list of the excluded measures [22,32,33,36-38]. Five reviews provided information on sample sizes [14,30-32,38] of the primary studies and three on the stage of stroke recovery when measurements were performed [14,30,31]. Although, the quality of the literature search and evaluation of psychometrics was generally high in the reviews, the methodology of the primary studies reporting these psychometric properties was not assessed using standardized checklists. This resulted in low total AMSTAR scores, ranging from 1 to 4 for all reviews (for exact scores, see Additional file 2).

### Extracted outcome measures

**Figure 2:**
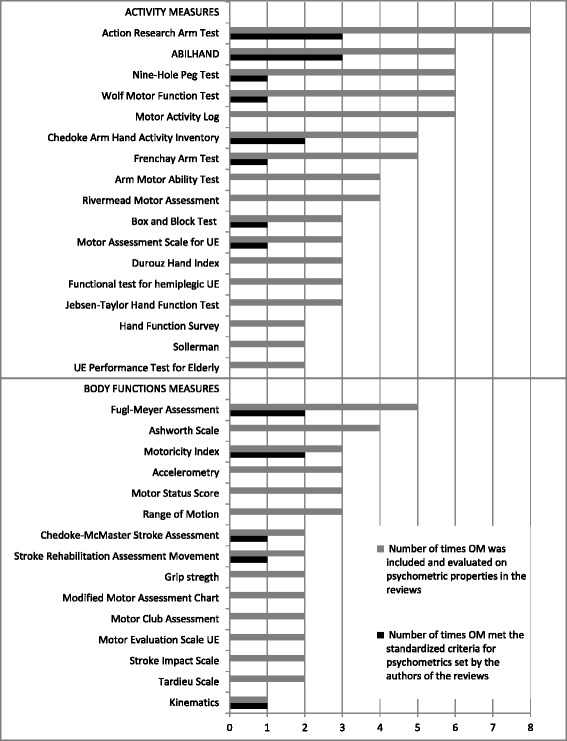
**Overview of outcome measures (OM) included in the reviews more than once (gray bars) and the number of times the OM met the criteria set for psychometric properties as reported in the reviews (black bars).**

In total, 53 different upper extremity related OM were included in the reviews, 31 were included in at least two reviews and eight in five or more studies (Table 2, Figure 2). Eight reviews provided sufficient information of the OM regarding psychometric properties (Table 2). From those eight reviews, 13 OM were identified that met the standards and criteria set for the psychometric properties by the authors of the reviews. The extracted information on psychometric properties and clinical utility as reported in the reviews is summarized in Tables 3 and 4. This final set of OM comprised five measures primarily targeting impairments of body functions and eight assessing limitations of activities. These OM cover a variety of OM assessing gross and fine motor function, muscle strength, objective movement analysis, dexterity, functional daily activities as well as self-reported arm and hand function. The detailed information on standards and criteria used in the reviews are summarized in Table 5.

**Table 3:** **Summary of psychometric properties and clinical utility of the outcome measures of impaired body function that met the standards or criteria set for the psychometric properties by the authors of the reviews**

	**FMA**		**MI-arm**	**CMSA**		**STREAM**	**Kinematics**
**References**	[32]	[14,38]	[32,37]	[32]	[14]	[21]	[14]
**Psychometric properties**							
Content validity						+	NA
Internal consistency		***				+	NA
Construct validity	+	***			***	+	**
Concurrent validity	+	***	+	+		+	
Floor/ceiling effect		**			NR		NR
Intra-rater, test-retest reliability	+	***	+		NR	+	***
Inter-rater reliability	+	***	+	+	***	+	
Responsiveness		**	*		NR		***
MCID, points		7p			NR		NR
MDC/SDD, points			12p				
**Clinical utility**							
Time to administer, minutes		20	10-15		60		varies
Administration burden		**			*		**

+Met the criteria set by the authors of the reviews (not graded); ***High/excellent/very good; **Moderate/good/adequate; *Low/poor; Administration burden includes: administration, scoring, interpretation and cost. *Abbreviations*: *NR* Not reported, *NA* Not applicable, *p* points, *s* seconds, *MCID* Minimal clinically important difference, *MDC* Minimal detectable change, *SDD* smallest detectable difference, empty space: not covered by the reviews; *FMA* Fugl-Meyer Assessment (motor), *MI* Motoricity Index, *CMSA* Chedoke-McMaster Stroke Assessment, *STREAM* Stroke Rehabilitation, Assessment Movement, FMA demonstrated a high level of measurement quality and clinical utility.

**Table 4:** **Summary of psychometric properties and clinical utility of the outcome measures of activity limitation that met the standards or criteria set for the psychometric properties by the authors of the reviews**

	**BBT**		**ARAT**		**CAHAI**		**WMFT**	**ABILHAND**		**FAT**		**MAS**		**NHPT**	
**References**	[31]	[14]	[31,32]	[14,35,38]	[21,23]	[14]	[14,38,35]	[21,22]	[14,35]	[37]	[14]	[32]	[14]	[32]	[14]
**Psychometric properties**															
Content validity					+			+							NA
Internal consistency				***	+		***	+							NA
Construct validity		***	+	**	+	***	***	+	**		NR	+	***	+	***
Concurrent validity	+		+		+		***	+		+		+		+	
Floor/ceiling effect		NR		*		NR	*		*		*		NR		NR
Intra-rater/test-retest reliability	+	***	+	***	+	NR	***	+		+	***		***	+	
Inter-rater reliability	+	***	+	***	+	***	***	+	***		***	+	***	+	***
Responsiveness		NR		**	+	***	**	+	**	*			NR		NR
MCID, points, seconds		6 blocks		6p		6.3p	12p		0.26-0.35 logits^a,d^				NR		32.8 s
				12-17p^c^			0.14-1.2p^a,b,c^								
MDC/SDD/SRD	+		+							1.3p					32.8 s
**Clinical utility**															
Time to administer, minutes		2		10		25	10-12		≥10	5-10			20-30		2
Administration burden		***		**		**	**		**		***		**		***

+Met the criteria set by the authors of the reviews (not graded); ***high/excellent/very good; **moderate/good/adequate; *low/poor; ^a^equal to effect size 0.2, ^b^clinical scale anchor, ^c^global rating, ^d^percentage of recovery (Simpson [35]); empty space, not covered by the reviews; Administration burden includes: administration, scoring, interpretation and cost. *Abbreviations*: *NA* Not applicable, *NR* not reported, MCID minimal clinically important difference, *MDC* minimal detectable change, *SDD* smallest detectable difference, *SRD* smallest real difference, *BBT* Box and Block Test, *ARAT* Action Research Arm Test, *CAHAI* Chedoke Arm Hand Activity Inventory, *WMFT* Wolf Motor Function Test, *FAT* Frenchay Arm Test, *MAS* Motor Assessment Scale, *NHPT* Nine Hole Peg Test, BBT, ARAT, CAHAI, WMFT, ABILHAND demonstrated a high level of measurement quality and clinical utility.

**Table 5:** **Standards and criteria for psychometrics and clinical utility provided by the authors of the reviews**

**Review**	**Criteria of psychometrics or clinical utility provided by the authors of the reviews**
Ashford [22]	Content validity, internal consistency, construct validity, test-retest reliability, agreement, responsiveness, interpretability: adequate design, method and results (Chronbach’s α: adequate 0.7-0.9, ICC: > 0.70); minimal clinically important difference presented, floor/ceiling effect ≤ 15%, time to administer < 10 min, administration burden: easy to sum up the items
Baker [21]	Psychometric testing have been performed
Connell [31]	Clinical utility criteria of ≥ 8points (time to administer and interpret ≤ 30 min, cost ≤ ₤ 100, simple equipment, portability), reliability/validity (kappa, correlation coefficients, ICC/r: strong ≥ 0.80, moderate 0.6-0.8, weak 0.4-0.6), ability to detect change (measurement error, standardized response mean, standardized error of measurement, limits of agreement, minimal detectable change)
Croarkin [32]	Significant correlations (p < 0.05) for test-retest, inter-rater reliability and validity (convergent, concurrent): level of evidence 1 = meets all 3 psychometrics criteria, level 2 = meets 2 of 3 criteria
Hillier [23]	Sound psychometrics: content and construct validity, reliability, sensitivity to change, utility (interpretability, acceptability, relevance)
Simpson [35]	MCID values calculated (related to effect size 0.2, anchor-based method using clinical scale, global rating, percentage of recovery)
Sivan [14]	Test-retest reliability (ICC/kappa: high/excellent ≥ 0.75, moderate 0.40-0.74, poor <0.40); internal consistency (Chronbach’s α: high/excellent > 0.80, adequate 0.70-0.79, low < 0.70); validity (correlation coefficient: excellent r > 0.60, adequate 0.30-0.59, poor <0.30), area under the curve: excellent > 0.90, adequate 0.70-0.89, poor < 0.70); responsiveness (effect size: large > 0.8, moderate 0.5-0.79, small <0.50; other adequate responsiveness methods, MCID value); floor/ceiling effect (excellent 0%, adequate < 20%, poor > 20%), respondent burden: (time, acceptance: excellent < 15 min, adequate: longer time, lower acceptability; poor: lengthy, acceptability problem); administrative burden (excellent: scoring by hand, easy to interpret, adequate: computer scoring, obscure interpretation; poor: costly, complex scoring/interpretation)
Tse [36]	Inter-rater, test-retest reliability (kappa/r/ICC > 0.75), internal consistency (Chronbach’s α > 0.80), content validity, construct validity (adequate method, r ≥ 0.60)
van Peppen [37]	Valid for stroke, test-retest reliability and concurrent validity (ICC/r ≥ 0.70), responsiveness (high/low), time to administer ≤ 15 min, test-protocol available: level of evidence 1 = meets all 6 criteria, level 2 = meets 5 of 6 criteria
Velstra [38]	Reliability (correlation coefficient, kappa, Chronbach’s α, ICC): very good or good/moderate; Responsiveness (effect size, standardized response mean): moderate or large

*Abbreviations*: *ICC* Intraclass coefficient, *MCID* minimal clinically important difference.

Among the final five OM targeting the impairments of body functions, the Fugl-Meyer Assessment (FMA, motor part) demonstrated the strongest level of psychometrics and clinical utility. For Motoricity Index (MI) the psychometrics are adequate except for responsiveness and for Chedoke-McMaster Stroke Assessment (CMSA) information is lacking concerning the responsiveness and the clinical utility is low. The stroke Rehabilitation Assessment of Movement (STREAM) and kinematic measures both demonstrated adequate psychometrics, but were only included and evaluated in one review, thus the information is still limited. Among the final eight OM assessing limitations of activities, a high level of psychometrics and clinical utility has been established for four capacity measures: Action Research Arm Test (ARAT), Box and Block Test (BBT), Chedoke Arm and Hand Activity Inventory (CAHAI), Wolf Motor Function Test (WMFT) and for a measure assessing perceived performance, ABILHAND. Responsiveness was low or information not provided for Frenchay Arm Test (FAT), Motor Assessment Scale (MAS) and Nine Hole Peg Test (NHPT). Thus, in total, six outcome measures (FMA, ARAT, BBT, CAHAI, WMFT and ABILHAND) demonstrated high level of measurement quality and clinical utility and can therefore be recommended for evaluation of upper extremity function and activity after stroke.

**Figure 3:**
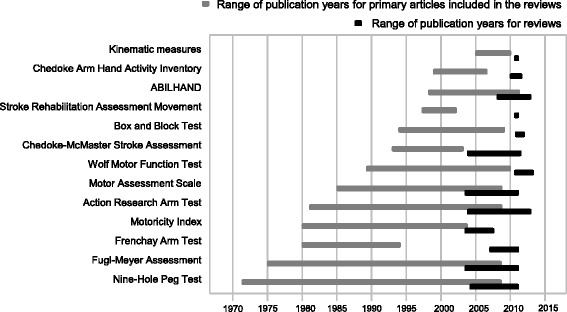
**Publication years for the primary references used in the systematic reviews and years when the reviews were performed, presented separately for every outcome measure included into the final set of measures.**

**Figure 4:**
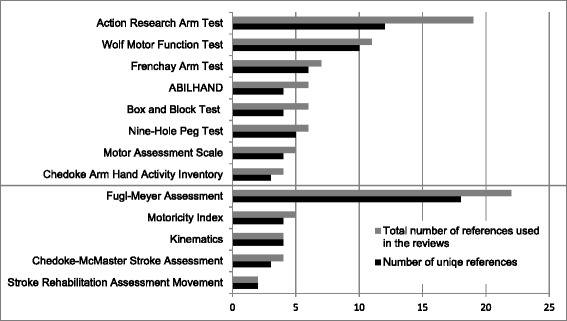
**Total number of references used in the reviews and number of references that were only used in one review (unique references) presented for outcome measures included into the final set.**

Figure 3 provides an overview of the publication years of the primary references used in the systematic reviews. Figure 4 illustrates the overlap of the primary articles included in the reviews, which was in average 19%. Accordingly, for nine OM none or only one primary source was used in more than one review, for BBT and ABILHAND 2 out of 6 primary articles were used in more than one review and for FMA 4 of 22 and for ARAT 7 of 19 references were used in more than one review.

## Discussion

The aim of this overview was to summarize and synthesize findings from single systematic reviews identifying the upper extremity outcome measures and evaluating the psychometric properties of these measures for stroke population. This approach enables a broader view of the research area and makes integration of findings possible between reviews using slightly different objectives or methodological process. In addition, unified findings from several reviews can provide a larger body of evidence and strengthen the recommendations based on these findings. In addition, areas were the evidence is lacking can be clarified and targeted in the future studies. To our knowledge, this overview is the first paper where the findings from multiple systematic reviews on upper extremity outcome measures has been summarized and integrated in a standardized way.

Several investigators have pointed out the importance of using clear and unified criteria for evaluation of the psychometric properties [16,21,39]. In the current overview, 8 of 13 reviews provided sufficient information regarding the psychometrics so that the OM meeting the criteria for psychometrics could be identified. Even when some variation between studies concerning the requirements set for the psychometrics could be observed, the differences were relatively small. In this overview, the psychometrics from different studies were integrated and the criteria used by the authors of the reviews are provided. This transparent reporting enables reasonable comparison between the findings from the reviews and also highlights the gaps in the research area.

In this overview of systematic reviews, 13 outcome measures met the standards and criteria set for the psychometric properties. Six of those, Fugl-Meyer Assessment (FMA), Action Research Arm Test (ARAT), Box and Block Test (BBT), Chedoke Arm and Hand Activity Inventory (CAHAI), Wolf Motor Function Test (WMFT) and ABILHAND, demonstrated a high level of measurement quality and clinical utility when information provided in the systematic reviews of psychometrics and clinical usefulness was taken into account. The psychometric properties of these six OM have been evaluated thoroughly in several studies. These OM are also widely used in research and in clinical practice and can be considered to have an acceptable and comparable clinical usability (administration, scoring, interpretation, cost and portability). Based on the findings of this overview, each of these six OM can be recommended to use for upper extremity assessment after stroke. However, when selecting a measure; the specific requirements of the study or clinical treatment goal, the expected outcome, the upper extremity disability level and the setting where the OM will be used, will need to be taken account. In addition, if several aspects of functioning are to be targeted, there is a need to include OM at different ICF levels [40,41].

Our findings, in general, are in line with recommendations made for OM in existing guidelines. All six recommended OM, apart from the CAHAI and ABILHAND, have also been recommended by the Dutch Clinical Practice Guideline for physical therapy in patients with stroke [12] or by the Neurology section of the American Physical Therapy Association (StrokeEdge task force) [13]. Both, CAHAI and ABILHAND are relatively new OM and this can be one possible reason why these have not yet been included in the guidelines. However, for two OM, the Frenchay Arm Test and the Motoricity Index, which both are recommended as basic OM in Dutch guidelines, the evidence reflected in the reviews regarding the responsiveness was not sufficient. As it can also be observed from Figure 3 in this overview, there is a clear lack of later studies, which can explain the poor reporting of responsiveness for these two OM. Nevertheless, the responsiveness is an important measurement property which needs to be considered when OM are selected for evaluation of treatment effects in clinical trials.

Findings from the current overview demonstrate that, at the body function level, the FMA was the only OM with sufficient evidence regarding the psychometric properties. This result suggests that the use of FMA (motor part) is warranted in research and in clinical practice. However, there is a need to improve and evaluate further other commonly used measures of motor function along with measures that evaluate other aspects of impairment, such as strength and range of motion. On the activity level, five capacity measures could be recommended. Among those, the BBT can be classified as fast screening tool for gross manual dexterity, providing information about the speed of performance, but offer no information on the reason of impaired performance or the quality of movement. The other three recommended capacity measures (ARAT, CAHAI, WMFT) are more time consuming and rely on the expertise of a therapist when the movement performance is scored. In general, these activity capacity measures assess the ability to perform functional tasks including lifting and moving of objects of various shapes and sizes. There is however some differences between these scales, e.g. in the ARAT the affected arm is assessed unilaterally, in the WMFT two bimanual items are tested, while in the CAHAI only bimanual tasks are assessed; in the WMFT the tasks are both timed (WMFT-time) and scored (functional ability score), while in the ARAT the time component is integrated into the different scoring levels; both in ARAT and WMFT the maximum score cannot be reached when compensatory movements are used for task completion in contrast to CAHAI where the independence and need of assistance is scored during the task performance. In addition to capacity measures, the ABILHAND can also be recommended to capture a person’s self-perceived manual performance during common daily life activities. One advantage using the ABILHAND is that it is validated using Rasch analysis method, which means that the score is expressed in logits and can be considered as an interval linear measure in statistical calculations [42]. It is however important to consider the implications of cognitive functions, such as memory and language deficits, which can significantly influence the validity of final score. Likewise, the ABILHAND questionnaire is most suitable in subacute and chronic phases of the stroke, when the person with stroke has some experience of performance difficulties during daily activities, so that these may be reliably scored.

In this overview, only reviews using systematic literature search were included in order to enable an unbiased selection of the outcome measures. There are, however, several publications available where detailed description on measurement properties for most common outcome measures is provided. Extensive work has been presented by the American Physical Therapy Association Neurology section task force, in which recommendations on OM were made for clinical practice, research and education. Four outcome measures, ARAT, BBT, FMA and WMFT recommended in this systematic overview were also recommended by this task force for use throughout different practice settings and during different stages of stroke recovery [13]. Another comprehensive overview of the psychometric and administrative properties of most common OM in stroke rehabilitation has also been provided by the Salter et al. [16,39]. Similar to our findings, it was concluded that reporting of the reliability and validity was relatively consistent across the scales, but less information was available on the responsiveness [16,39].

The majority of the OM extracted into the final list are well-established observational clinical scales using ordinal scoring. Even though the good psychometric properties have been demonstrated for these scales, the ordinal scoring is considered to be less sensitive to change compared to continuous scales and highly dependent on the observer and the pre-set scoring levels. The disadvantage of both observational scales and timed testing such as NHPT, BBT is that the qualitative detailed information of movement performance and motor compensation strategies are not fully captured [43]. To capture these movement qualities a more sophisticated, detailed and potentially technological assessments are often required. Kinematic movement analysis, which also was identified in one of the reviews, is one such method that allows a more detailed analysis of movements. Today, the most established method for kinematic analysis is the optoelectronic motion capture systems. However, considering the cost, availability, and knowledge needed for this kind of movement analysis, the main application area will predominantly remain to be in the research.

The current rapid development of technologies, such as inertial sensors, providing kinematic data on upper extremity use in daily life both inside and outside the laboratory is exciting. There is opportunity to extend the quality and accuracy of measurement, filling the gaps not covered by the more traditional clinical scales. However, there are variations between the different technology-assisted systems and no standardized guidelines or test procedures have been established. This was also observed in this overview, where two reviews incorporated accelerometer-based assessments, with no standardized evaluation on psychometric properties provided by the authors of the reviews. The instrumented testing, where the performance during a clinical assessment is complemented with simultaneous acquisition of movement data gathered from motion sensors, virtual reality applications or instrumented objects, is another promising method that provides a more objective clinical assessment [44-47]. For the patient reported measures, the computerized adapted testing is also emerging and would considerably decrease the patient and administrator burden for information collection [48,49]. More research is, however, needed regarding the benefits of these instrumented assessment tools and their psychometric properties.

### Limitations and strengths

Our approach to integrate findings from multiple reviews has some limitations. First, the conclusions are based on the information provided by the authors of the reviews and data from primary studies was not retrieved or evaluated. Second, it could be expected that there would be some overlap of primary articles used for reporting the psychometric properties of outcome measures in the reviews. This overlap was, however, small (<20%) in the current systematic overview. For the majority of outcome measures none or only one primary source was used in more than one review. A larger overlap was only observed for two OM (ARAT, FMA) in which also the number of original articles was larger.

In the current overview the reviews older than ten years were not included. This decision was based on the argument that there has been a shift of paradigm regarding the evaluation of the psychometric properties of the measurement scales. The requirements on appropriate statistical methods and interpretation of the results have been changed compared to earlier studies. The intention was to capture information of up-to-date measurement scales. It is also evident that recently developed newer OM, including technology-assisted OM, have not been included in the reviews and are subsequently not captured in the current overview. Findings from this overview showed also that the methodological quality of the included systematic reviews was relatively high regarding the literature search and the study selection, but limited information was provided concerning the primary studies where the outcome measures were extracted from. To improve critical appraisal of the methodological quality in primary studies, validated checklists and standards should be used in the future reviews. One such checklist has been developed for the health-related patient-reported outcomes (COSMIN-checklist), which can give some guidance also for evaluation of other measurement instruments, such as clinical rating scales [50].

## Conclusions

This overview of systematic reviews provides a comprehensive systematic synthesis of evidence regarding the psychometric properties and clinical utility of the upper extremity outcome measures after stroke. The findings from this overview can provide guidance for clinicians, researchers and policy makers for evidence-based outcome measure selection. Altogether, thirteen outcome measures met the standards and criteria set for the psychometric properties and six of those demonstrated a high level of measurement quality and clinical utility. The Fugl-Meyer Assessment (FMA) on body function level and the Action Research Arm Test (ARAT), Box and Block Test (BBT), Chedoke Arm and Hand Activity Inventory (CAHAI), Wolf Motor Function Test (WMFT) and ABILHAND on activity level cover a broad spectrum of assessments and can be recommended for assessment of upper extremity function and activity in research and clinical praxis. Future research needs to investigate the psychometric properties of other commonly used OM on body function level and the upcoming technology-supported upper extremity measures.

## Additional files

Additional file 1:
**Excluded reviews from full-text screening and the reason for exclusion.**
Additional file 2:
**Summary of the assessment of the methodological quality of the reviews using the AMSTAR (Assessment of Multiple Systematic Reviews) tool.**

## Abbreviations

AMAT: Arm motor ability test
ARAT: Action research arm test
BBT: Box and block test
CAHAI: Chedoke arm and hand activity inventory
CMSA: Chedoke-Mcmaster stroke assessment
DHI: Durouz hand index
FAT: Frenchay arm test
FMA: Fugl-Meyer assessment
HAST: Hand active sensation test
JHFT: Jebsen-Taylor hand function test
MAL: Motor activity log
MAS: Motor assessment scale
MCA: Motor club assessment
MESUPE: Motor evaluation scale upper extremity
MI: Motoricity index
MFT: Manual function test
MMAC: Modified motor assessment chart
MRC: Medical Research Council
MSS: Motor status score
NHPT: Nine-Hole Peg Test
RMA: Rivermead motor assessment
ROM: Range of motion
SIS: Stroke impact scale
STREAM: Stroke rehabilitation assessment of movement
TEMPA: Upper extremity performance test for elderly
UBDS: Upper body dressing scale
UMAQS: University of Maryland Arm Questionnaire for Stroke
VAS: Visual analog scale
WMFT: Wolf motor function test
