# Supplementary material for: An overview of systematic reviews on upper extremity outcome measures after stroke
Source: BMC Neurol. 2015 Mar 11;15:29. doi: 10.1186/s12883-015-0292-6 (PMC4359448; doi:10.1186/s12883-015-0292-6)
Supplement: Additional file 1: — Excluded reviews from full-text screening and the reason for exclusion. [file 12883_2015_292_MOESM1_ESM.docx]

Additional file 1. Excluded reviews from full-text screening and the reason for exclusion

| **No.** | **Author year** | **1^st^ reason** | **Supplementary information** |
| --- | --- | --- | --- |
| 1 | Asford, 2013 | Treatment effect | Psychometrics not described or assessed |
| 2 | Balu, 2009 | Treatment effect | Psychometrics no described or assessed |
| 3 | Barak, 2006 | Educational review |  |
| 4 | Chen, 2009 | Identify predictors of recovery |  |
| 5 | Duncan, 2013 | Educational review | Full-text not available |
| 6 | Haugh, 2006 | Selected OM, Tardieu | Systematic literature search on selected OM, no criteria |
| 7 | Kasner, 2006 | Educational review |  |
| 8 | Kelly-Hayes, 2004 | Educational review | Full-text not available |
| 9 | Kitsos, 2011 | Selected OM, validity | Literature search on most frequently used OM, criteria on systematic review |
| 10 | Kücükdeveci,2011 | Educational review |  |
| 11 | Lang, 2013 | Educational review | Psychometrics reported for commonly used OM, no criteria |
| 12 | Lawrence, 2011 | Qualitative studies | Psychometrics not reviewed |
| 13 | Lin, 2009 | Not review |  |
| 14 | Magasi, 2010 | No UE measures included |  |
| 15 | Marciniak, 2011 | Educational review |  |
| 16 | Peter, 2011 | Psychometrics no described or assessed |  |
| 17 | Rowland, 2008 | Selected OM, OT | Systematic literature search on selected OM, criteria on # of studies |
| 18 | Salter, 2005 | Selected OM, Body function | Literature search on selected OM, criteria |
| 19 | Salter, 2005 | Selected OM, Activity | Literature search on selected OM, criteria |
| 20 | Salter, 2005 | Selected OM, Participation | Literature search on selected OM, criteria |
| 21 | Salter, 2007 | Psychometrics no described or assessed |  |
| 22 | Sullivan, 2013 | No review, case report | Psychometrics not described |
