# Supplementary material for: An overview of systematic reviews on upper extremity outcome measures after stroke
Source: BMC Neurol. 2015 Mar 11;15:29. doi: 10.1186/s12883-015-0292-6 (PMC4359448; doi:10.1186/s12883-015-0292-6)
Supplement: Additional file 2: — Summary of the assessment of the methodological quality of the reviews using the AMSTAR (Assessment of Multiple Systematic Reviews) tool. [file 12883_2015_292_MOESM2_ESM.docx]

Additional file 2. Summary of the assessment of the methodological quality of the reviews using the AMSTAR (Assessment of Multiple Systematic Reviews) tool.

| AMSTAR items | Ashford  2008 | Baker  2011 | Connell  2012 | Croakin  2004 | Gebrues  2010 | Hillier  2010 | Lemmens  2012 | Platz  2005 | Simpson  2013 | Sivan  2011 | Tse  2013 | Peppen  2007 | Velstra  2011 | n |
| --- | --- | --- | --- | --- | --- | --- | --- | --- | --- | --- | --- | --- | --- | --- |
| 1. Was a priori design provided? | - | - | - | - | - | - | - | - | - | - | - | - | - |  |
| 2. Duplicate study selection and data extraction? | √ | - | √ | ? | ? | ? | ? | √ | - | √ | - | √ | ? | 5 |
| 3. Was a comprehensive literature search performed? | √ | √ | √ | √ | √ | √ | √ | √ | √ | √ | √ | √ | √ | 13 |
| 4. Was the status of publication (i.e. grey literature) used as an inclusion criterion? | √ | - | ? | ? | - | - | ? | √ | √ | ? | - | √ | √ | 5 |
| 5. Was a list of studies (included and excluded) provided? | - | - | - | - | - | - | - | - | - | - | - | - | - | 0 |
| 6. Were the characteristics of the included studies provided? | - | - | - | - | √ | - | - | - | - | √ | - | - | - | 2 |
| 7. Was the scientific quality of the included studies assessed and documented? | - | - | - | - | - | - | - | - | - | - | - | - | - | 1 |
| 8. Was the scientific quality of the included studies used appropriately in formulating conclusions? | - | - | - | - | - | - | - | - | - | - | - | - | - | 1 |
| 9. Were the methods used to combine the findings of studies appropriate? | - | - | - | - | - | - | - | - | √ | - | - | - | - | 1 |
| 10. Was the likelihood of publication bias assessed? | - | - | - | - | - | - | - | - | - | - | - | - | - | 0 |
| 11. Was the conflict of interest included? | - | √ | √ | - | √ | - | √ | - | √ | - | - | - | √ | 6 |
| Total AMSTAR score | 3 | 2 | 3 | 1 | 3 | 1 | 2 | 3 | 4 | 3 | 1 | 3 | 3 |  |
